# Navigating the contested borders between myelodysplastic syndrome and acute myeloid leukemia

**DOI:** 10.3389/fonc.2022.1033534

**Published:** 2022-10-28

**Authors:** Alexander J. Ambinder, Amy E. DeZern

**Affiliations:** Department of Oncology, Sidney Kimmel Comprehensive Cancer Center, Johns Hopkins University School of Medicine, Baltimore, MD, United States

**Keywords:** myelodysplastic syndromes, acute myeloid leukemia, hematologic malignancies, transformation, secondary AML, clonal hematopoiesis

## Abstract

Myelodysplastic syndrome and acute myeloid leukemia are heterogeneous myeloid neoplasms which arise from the accumulation of mutations in a myeloid stem cell or progenitor that confer survival or growth advantages. These disease processes are formally differentiated by clinical, laboratory, and morphological presentations, especially with regard to the preponderance of blasts in the peripheral blood or bone marrow (AML); however, they are closely associated through their shared lineage as well as their existence on a spectrum with some cases of MDS displaying increased blasts, a feature that reflects more AML-like behavior, and the propensity for MDS to transform into AML. It is increasingly recognized that the distinctions between these two entities result from the divergent patterns of genetic alterations that drive each of them. Mutations in genes related to chromatin-remodeling and the spliceosome are seen in both MDS and AML arising out of antecedent MDS, while mutations in genes related to signaling pathways such as *RAS* or *FLT3* are more typically seen in AML or otherwise are a harbinger of transformation. In this review, we focus on the insights into the biological and genetic distinctions and similarities between MDS and AML that are now used to refine clinical prognostication, guide disease management, and to inform development of novel therapeutic approaches.

## Introduction

Myelodysplastic neoplasms (MDS) and acute myeloid leukemia (AML) are two disease entities that together form a spectrum of myeloid neoplasms with a common pathogenesis. The diseases are linked by similarities in their biology, clinical presentation, management, and through the capacity of MDS to transform into AML in the setting of clonal evolution or progression. The main risk factor for both diseases is advanced age, though they may also be predisposed by environmental exposures, and in a minority of cases, germline mutations (1–3). However, at their extremes, these disease entities are fundamentally distinct and can have dramatically different clinical presentations and prognoses. The approach to expectation management for an individual patient as well as therapeutic planning may vary between MDS and AML as well as within each individual disease. The two diseases can be challenging to distinguish and also overlap in ways that may lead clinicians to treat with one disease-specific paradigm when in fact the biology calls for another, highlighting the importance of a nuanced understanding of their association.

The notion that leukemia could be preceded by a pre-leukemic bone marrow failure state characterized by cytopenias and abnormal morphology began coalescing in the mid-1900s, nearly 100 years after acute leukemia was first described (4, 5). Even then, the distinctions between the numerous disease states that fall under the umbrella of bone marrow failure were poorly defined. It wasn’t until 1970 that the term myelodysplastic syndrome (now referred to as myelodysplastic neoplasms) was first introduced to distinguish it from other bone marrow failure states.

A key feature that distinguished MDS from other bone marrow failure states was the observation in the 1980s that, like AML, MDS is a clonal process (6). This insight was first deduced from a study in the 1980s that showed skewed X-chromosome inactivation mosaicism in the bone marrow of a patient with MDS. It was subsequently determined that up to 50% of cases of MDS have cytogenetic abnormalities indicative of clonality (7). When combined with targeted genetic sequencing, nearly 90% of patients are found to have a clonal abnormality, and with research techniques including whole exome sequencing, genetic abnormalities indicative of clonality can be identified in virtually all cases (8–11). MDS has thus come to be defined by the World Health Organization (WHO) as a clonal disease process of hematopoietic precursors that exhibits abnormal morphology (i.e. dysplasia), leads to ineffective hematopoiesis leading to cytopenias, and has the potential to transform into AML (12).

## The pathogenesis of MDS and AML

Understanding the connection between MDS and AML hinges on an understanding of their shared pathogenesis. MDS and AML both arise through a process of clonal evolution in which the sequential acquisition of selectively advantageous mutations leads to clonal dominance, and eventually, malignant behavior. These mutations result in clonal hematopoiesis of indeterminate potential (CHIP), in which the mutated clone predominates over unmutated clones but doesn’t produce ineffective hematopoiesis or overt neoplasia as seen in MDS and AML (13–17). Although not considered malignant, CHIP may not be clinically silent, as it has been associated with a variety of non-hematologic disease states (18, 19), most notably atherosclerosis and myocardial infarction (15, 20).

CHIP is associated with an increased risk for progression to a hematologic malignancy, though only a minority of patients will develop overt malignancy (17). Some CHIP-associated mutations are more highly associated with progression to malignancy than others (21). Among those who do progress, the onset of CHIP may precede the hematologic malignancy by years or decades. An intermediary entity known as clonal cytopenias of undetermined significance (CCUS), defined by the presence of somatic mutations indicative of clonal hematopoiesis accompanied by clinically significant cytopenias, has been well described and represents a transitional state between CHIP and overt malignancy (22). CHIP and CCUS have different genetic features; CCUS is more likely to bear mutations in genes such as U2AF1, ZRSR2, SRSF2, JAK2, and RUNX1. CCUS is also more likely to harbor multiple co-incident genetic mutations and the mutations tend to be found at higher variant allele frequencies (VAFs) (21). This is not to say that CHIP cannot have any of these features, but that the acquisition of these features correlates with the development of cytopenias leading to its redefinition as CCUS. Unsurprisingly, patients with CCUS are at higher risk of developing overt malignancy (23). Since CHIP represents the first step on the path to leukemogenesis, it is also unsurprising that the same risk factors that predispose to MDS and AML (e.g. age, inflammation, DNA-damaging chemotherapies, and radiation) also promote the development and increase selective pressure for CHIP (24).

**Figure 1:**
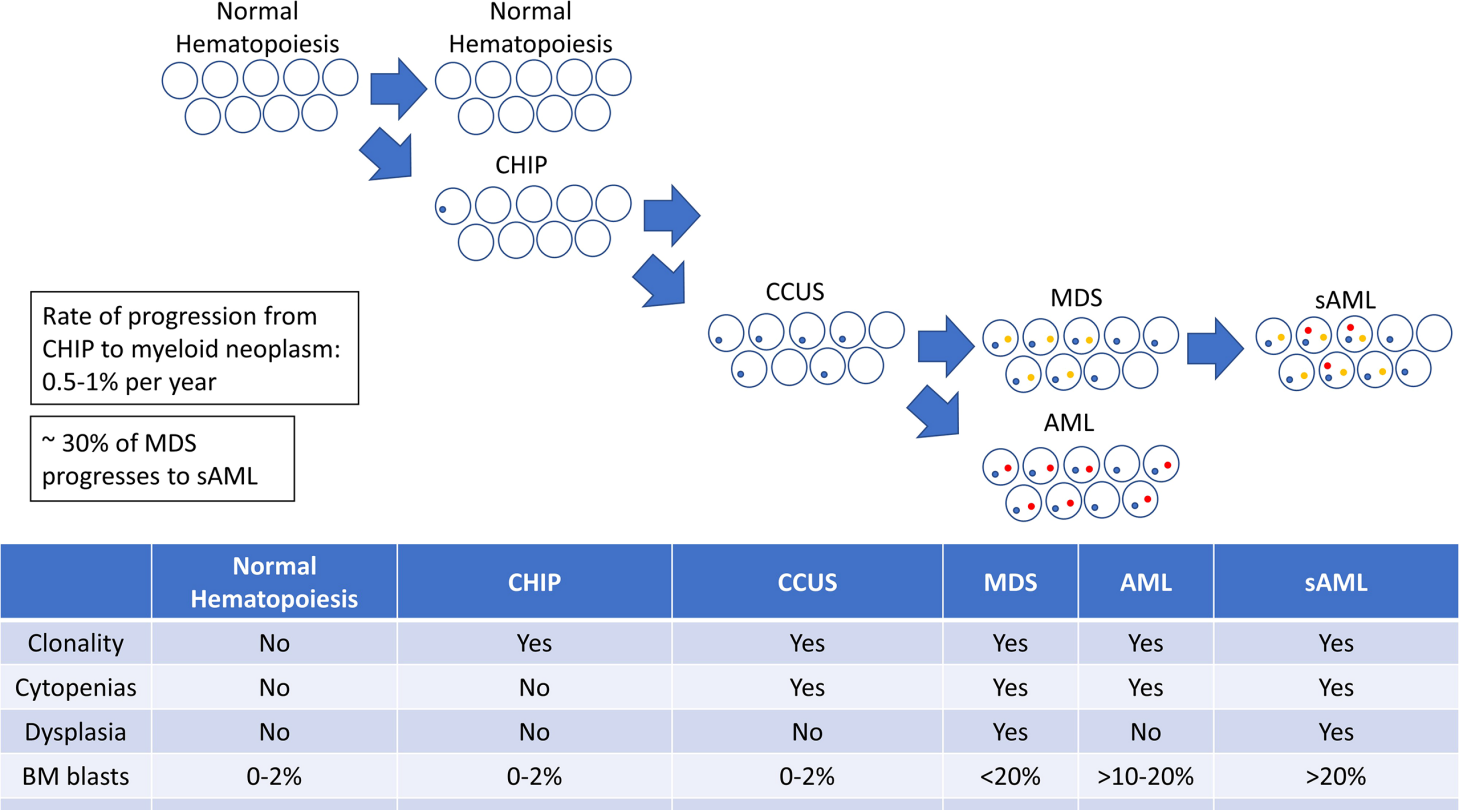
A diagram illustrating the progression from clonal hematopoiesis to MDS, AML, and sAML.

Expansion or evolution of the hematopoietic clone in CHIP or CCUS then leads to a progressively increased risk of developing a myeloid malignancy. The complex interplay of the underlying genetic and epigenetic changes determines the phenotype of the disease with respect to the MDS-AML spectrum. Additional genetic changes may drive further clonal and phenotypic evolution, resulting in the transformation of one disease phenotype (i.e. MDS) to another (i.e. AML) (**Figure 1**).

## The genetic landscape of myeloid malignancies

Broadly, the genes that are implicated in myeloid clonal evolution and leukemogenesis are involved in a narrow set of cellular functions: *DNMT3A*, *TET2*, *IDH1/2* are involved in the coordination of DNA methylation; *ASXL1*, *BCOR*, and *EZH2* are involved in histone modification; *SF3B1*, *SRSF2*, *ZRSR2*, and *U2AF1* encode components of the spliceosome; *STAG2*, *SMC3*, *SMC1A*, and *RAD21* form components of the cohesin complex; *RUNX1*, *GATA2*, and *ETV6* are transcription factors; *TP53* is a tumor suppressor gene; and *JAK2, NRAS, KRAS, FLT3, WT1, NF1, CBL*, and *PTPN11* are all involved in cell signaling. With some exceptions, mutations infrequently occur in multiple genes of the same category within the same clone. This is exemplified by the rarity of mutations in multiple spliceosome genes (9) and the relative exclusivity of mutant *TET2* and mutant *IDH2*. There is little advantage to accumulating additional mutations within a pathway once it has already been disrupted (or activated). Conversely, mutations in genes from multiple categories are often required for malignant transformation (25–28).

Among the most commonly mutated genes in CHIP are *DNMT3A, ASXL1, TET2, JAK2, TP53, PPM1D, SRSF2*, and *SF3B1* (13, 15, 16 ,29). *DNMT3A* and *TET2* may be somatically mutated in MDS and AML, suggesting that the acquisition of these mutations is part of a common pathway in the development of both diseases; in these cases, it may be the specific subsequent mutations that determine whether a patient develops MDS or AML. Mutations in other genes such as *SF3B1* and *SRSF2*, however, are more commonly found in MDS and MDS/AML, indicating that cases of CHIP bearing these mutations may be skewed towards the development of MDS over AML at an early stage in clonal evolution (9). Founder mutations also synergize with specific subsequent mutations to promote clonal advantage. Therefore, one mutation predisposes to the acquisition of another specific mutation. In this manner, a founder mutation sets in motion a stereotyped cascade of mutational events leading to a predictable disease phenotype (9).

Cancer is a clonal phenomenon, but multiple subclones may emerge in the course of disease evolution, as is particularly evident in the case of MDS. Multiple subclones, united by a shared, common clonal ancestor and distinguished by unique genetic mutations that have accumulated since then, exist in competitive stasis within the MDS bone marrow (30). These subclones span the gamut of residual healthy bone marrow, dysplastic elements with limited residual capacity for hematopoiesis, and myeloblasts, which have experienced a complete arrest of maturation and therefore make no contribution to hematopoiesis. This is reflected in the high allelic burden of MDS-associated somatic mutations and the relatively low bone marrow blast percentage. The opportunity for any individual subclone to acquire new, advantageous mutations within this environment results in the stochastic nature of MDS.

## MDS and AML are biologically distinct

Despite similarities in their origins, MDS and AML, at their extremes, are biologically quite distinct. AML is a proliferative neoplasm that produces cytopenias through blocks in differentiation that prevent leukemic blasts from maturing and by displacing normal hematopoietic elements. In contrast, low risk MDS leads to cytopenias through dysfunctional maturation and increased rates of cell turnover and death in the neoplastic cells. Furthermore, neoplastic cell death through inflammatory (pyroptosis and necroptosis) mechanisms generates a highly inflammatory bone marrow microenvironment, which stifles normal hematopoiesis and drives further clonal evolution (31, 32). Thus, most of the hematopoietic cells in circulation in patients with MDS are actually derived from the neoplastic clone, though they may be abnormal and insufficient in quantity and function. The mechanistic differences between the cytopenias observed in AML and MDS have important implications for the hematologic effects of anti-neoplastic therapies. Whereas cytotoxic therapies that eliminate leukemic blasts in AML can restore normal hematopoiesis, the effect in MDS is the destruction of the bulk of the residual hematopoietic (albeit dysfunction) elements.

## MDS and AML are genetically distinct

The biological and phenotypic differences between MDS and AML are underpinned by genetic differences.

**Figure 2:**
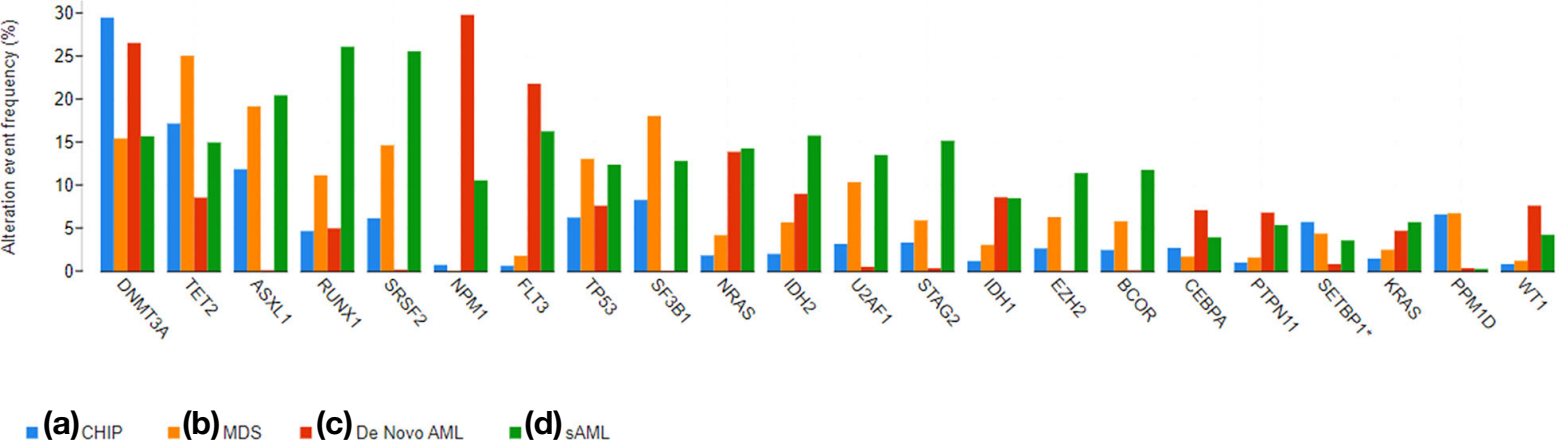
A bar graph demonstrating the prevalence of commonly mutated genes in CHIP, MDS, *De Novo* AML, and sAML. The graphs were generated using cBioPortal (33, 34) and data from the referenced datasets (25, 35–38).

**Figure 2** shows the distribution of mutations in MDS, AML without antecedent myeloid neoplasm (*de novo* AML), and AML with antecedent myeloid neoplasm (secondary AML, sAML) (7, 39). While mutations such as DNMT3A and TET2 may be seen in all three entities, unbalanced chromosomal abnormalities and mutations in genes involved in the spliceosome are seen in both MDS and sAML (9, 40). These MDS-specific mutations produce the MDS phenotype. Deletion 5q is one of the most common mutations in MDS and when it is present in relative isolation, it is associated with the so-called 5q minus syndrome, an indolent form of MDS with a predominant anemia and sometimes, thrombocytosis. In mouse models, deletion of individual genes on the short arm of chromosome 5 including *RPS14, HSPA9* and *CD74, miRNA-145, Fli1*, and *CSNK1A1*, recapitulate the macrocytic anemia, neutropenia, and thrombocytosis observed in 5q minus syndrome (41).

MDS with mutations in *SF3B1* is another example in which the specific mutation correlates closely with the presence of ring sideroblasts. *SF3B1* is seen in approximately 80% of patients with MDS with ring sideroblasts (MDS-RS) and single lineage dysplasia and 40% of those with MDS-RS with multilineage dysplasia (35, 42–44). Furthermore, the *SF3B1* mutant variant allele frequency (VAF) correlates with the burden of ring sideroblasts in the marrow. It is not entirely clear how *SF3B1* mutation produces the RS phenotype (45), however, knock out or inhibition of *SF3B1* in mouse models and *in vitro* also results in the development of RS, confirming the causative association (46).

While deletion 5q and *SF3B1* mutation most clearly demonstrate the relationship of genotype to phenotype, there are a variety of other mutations that are associated with an MDS phenotype including idic(X)(q13), isochromosome 17q, 17p deletion or loss of 17p, monosomy 13 or 13q deletion, 12p deletion or loss of 12p, 11q deletion, monosomy 7, 7q deletion, or loss of 7q, complex karyotype, and pathologic mutations in *ASXL1, BCOR, EZH2, SRSF2, STAG2, U2AF1*, and *ZRSR2* (8, 12). Common themes in the mutational profile of MDS are the loss of chromosomal material, unbalanced translocations, and spliceosome mutations (*SF3B1, SRSF2, ZRSR2*, and *U2AF1*). While some of the mutations in the spliceosome genes are hotspot mutations that may alter function, many of the MDS-associated mutations lead to loss of function and are therefore less amenable to drug targeting.

*De novo* AML, on the other hand, frequently bears mutations in genes such as *FLT3, NPM1, IDH1/2, CEBPA*, *WT1, PTPN11*, and *KRAS* (16). These mutations tend to be activating and many of the genes are involved in signaling pathways (*FLT3, NPM1, WT1, PTPN11, KRAS*), thus leading to a proliferative phenotype (47). This proliferative phenotype renders *de novo* AML more sensitive to conventional cytotoxic chemotherapy agents and the activating mutations provide more opportunities for pharmaceutical inhibition.

Secondary AML is a disease process in which MDS has undergone further clonal evolution and acquired additional mutations that lead to the development of an AML phenotype. SAMLs bear gene mutations that are frequently seen in MDS, but also have additional genetic mutations in genes that are associated with AML such as *NRAS, FLT3, WT1, NPM1, IDH1/2*, and *PTPN11* (48, 49). The presence of MDS-associated chromosomal abnormalities or somatic gene mutations are actually specific to sAML and distinguish it from *de novo* AML. The WHO classification system has included the presence of MDS-associated chromosomal abnormalities as a defining characteristic of sAML for some time, however it is only in the most recent versions of the WHO and ICC that specific somatic mutations were included in the definition. This change is based largely on results from a study comparing the genetic profiles of *de novo* AML and rigorously clinically defined sAML in which the presence of mutations *SRSF2,SF3B1, U2AF1, ZRSR2, ASXL1, EZH2, BCOR*, or *STAG2* was highly specific for a diagnosis of sAML (50). Furthermore, the investigators identified a subset of patients who were either elderly or had therapy-related AML who had clinically diagnosed *de novo* AML, but had genetic profiles and clinical outcomes most consistent with sAML. Biologically and behaviorally, their disease was sAML, even if an antecedent myeloid neoplasm had not been formally diagnosed.

## The evolution of myeloid neoplasm classification

Historically, the presence of blasts in the peripheral blood or bone marrow has been a defining feature of AML, and blast percentage has been one of the key features used to distinguish MDS from AML. The first efforts to define and classify AML and later, MDS, were undertaken by the French-American-British (FAB) group. AML was defined by the presence of ≥30% blasts, whereas MDS was defined according to the percentage of blasts, the presence of ring sideroblasts, and the presence of monocytes (51). The categories included: refractory anemia (<5% blasts, <15% ring sideroblasts), refractory anemia with ring sideroblasts (≥ 15% ring sideroblasts, < 5% marrow blasts), refractory anemia with excess blasts (5-19% blasts in the marrow and 1-5% in the peripheral blood), refractory anemia with excess blasts in transformation (20-29% marrow blasts, > 5% peripheral blood blasts), and chronic myelomonocytic leukemia (absolute monocyte count in the peripheral blood > 1000).

Despite the reality that the FAB system is now mostly considered in an historical fashion, it did highlight two important features of the natural history of MDS that remain relevant today: first, that cases with higher percentages of blasts were more likely to progress and transform into overt AML, an association that was captured by other prognostic scoring systems (52); second, some cases never progress to acute leukemia, yet still result in significant disease chronicity, tremendous morbidity and eventual mortality attributable to MDS. It is now known that approximately 20-30% of cases of MDS progress to overt AML while approximately 30% of cases of AML are thought to arise out of an antecedent hematologic disorder such as MDS (53).

## Updates in the classification of MDS and AML

The advent of next generation sequencing has led to an ongoing process of redefining the borders of MDS, AML, and the subtypes within each of them. **Tables 1**, **2** compare and contrast the most recent classification systems from the World Health Organization (WHO) and the International Consensus Classification for MDS and AML, respectively. The most recent iterations of MDS and AML classification systems emphasize genetic factors over morphological and clinical features in defining disease subtypes. In the WHO classification schemas, MDS and AML are first divided into those with defining genetic abnormalities and those without. It is only those subtypes without genetically defining features that are further characterized according to morphology. The same genetically defined MDS and AML subtypes are also included in the ICC classification schemas, however, in the case of MDS with ring sideroblasts and AML as a whole, the ICC goes even further and discards morphological classification altogether.

**Table 1:** A comparison of the WHO and ICC classification systems for MDS. Criteria specific to the ICC classification system are color coded red. Classification of Myelodysplastic Neoplasms (MDS)^1,2,3^.

WHO 2022^2^	ICC^3^	Dysplastic lineages	Cytopenias	Cytoses^4^	Blasts		Cytogenetics	Mutations		Diagnostic Qualifiers^5^
MDS with defining genetic abnormality										Therapy-related or Germline Predipsosition
MDS-5q (low blasts and isolated 5q deletion (MDS-5q))	MDS with del(5q) [MDSdel(5q)]	Typically >1^6^	≥1	Thromobcytosis allowed	<5% BM, <2% PB		5q deletion alone, or with 1 other abnormality other than del 7 or del 7q	Any, except multi-hit TP53		
MDS with low blasts and SF3B1 mutation (MDS-SF3B1)					<5% BM, <2% PB		Absence of 5q deletion, del 7, deletion abn3q26.2 or complex karyotype	SF3B1	( ≥15% ring sideroblasts (RS) may substitute for SF3B1 mutation)	
	MDS with mutated SF3B1 (MDS-SF3B1)	Typically >1^6^	≥1	0					*(≥10% VAF), without multi-hit TP53, or RUNX1*	
MDS with biallelic TP53 inactivation (MDS-bi*TP53)*	MDS with mutated TP53	–	Any	–	<20% BM, PB	0-9% BM or PB	Usually complex	2 or more *TP53* mutations, or 1 mutation with evidence of *TP53* copy number loss or cnLOH at the 17p TP53 locus	Multi-hit TP53 mutation^7^, or TP53 mutation (VAF >10%) and complex karyotype often with loss of 17p^8^	
	MDS/AML with mutated TP53	–	Any	–		10-19% BM or PB			Any somatic TP53 mutation (VAF >10%)	
MDS, morphologically defined										
MDS with low blasts (MDS-LB)	MDS, NOS - without dysplasia	0	≥1	0	<5% BM, 2-4% PB			Any, except multi-hit TP53 or SF3B1 (≥ 10% VAF)		
	MDS, NOS - with single lineage dysplasia	1	≥1	0				Any, except multi-hit TP53; not meeting criteria for MDS-SF3B1		
	MDS, NOS - with multilineage dysplasia	≥2	≥1	0				Any, except multi-hit TP53; not meeting criteria for MDS-SF3B1		
MDS, hypoplastic (MDS-h)^9^	–				<5% BM, 2-4% PB					
MDS with increased blasts (MDS-IB)										
MDS-IB1	MDS with excess blasts (MDS-EB)	Typically >1^6^	≥1	0	5-9% BM or 2-4% PB			Any, except multi-hit TP53		
MDS-IB2^10^	MDS/AML	Typically >1^6^	≥1	0	10-19% BM or 5-19% PB, Auer rods (Age ≥ 18, not pediatric)		Any, except AML- defining	Any, except NPM1, bZIP CEBPA or TP53		
MDS with fibrosis (MDS-f)	–				5-19% BM, 2-19% PB					

^1^ Defined by cytopenias and dysplasia (≥10% for all lineages). In general, there should be clinical evidence that the blood count abnormality is chronic in duration (typically 2-4 months or longer), and is not explained by a drug, toxin, or comorbid condition.

^2^ Khoury J, et al, 5th Edition WHO Classification of Haematolymphoid Tumours: Myeloid and Histiocytic/Dendritic Neoplasms. Leukemia 2022.

^3^ Arber D, et al, The International Consensus Classification (ICC) of Myeloid Neoplasms and Acute Leukemias: Integrating Morphological, Clinical, and Genomic Data. Blood 6/14/2022.

^4^ Cytoses: Sustained white blood count ≥13x109/L, monocytosis (≥5x109/L and ≥% of leukocytes), or platelets ≥450x109/L; thrombocytosis is allowed in MDS-del(5q) or in any MDScase with inv(3) or t(3;3) cytogenetic abnormality.

^5^ Therapy-relatedness and underlying germline predisposition conditions are applied as qualifiers to the diagnosis.

^6^ Although dysplasia is typically present in these entities, it is not required.

^7^ Defined as two distinct TP53 mutations (each VAF>10%) OR a single TP53 mutation with either 1) 17p deletion on cytogenetics; 2) VAF of >50%; or 3) Copy-neutral loss of heterozygosity (LOH) at the 17p TP53 locus.

^8^ If TP53 locus LOH information is not available.

^9^ ≤25% bone marrow cellularity, age adjusted.

^10^ MDS-IB2 (MDS/AML) may be regarded as AML-equivalent for therapeutic considerations and from a clinical trial design perspective when appropriate.

**Table 2:** A comparison of the WHO, ICC, and ELN 2022 classification systems for AML. Criteria specific to the ICC, ELN 2022, or both classification systems are color coded red, blue, and purple, respectively. Classification of Acute Myeloid Leukemias (AML)^1,2,3^.

WHO 2022^1^	ICC^2^	Blasts		Cytogenetics	Mutations	Differentiation	Diagnostic Qualifiers^4^
Acute myeloid leukaemia with defining genetic abnormalities							Therapy-related, progressing from myelodysplastic syndrome, progressing from myelodysplastic/myeloproliferative neoplasm (specify), germline predisposition
APL with *PML*::*RARA* fusion	APL with t(15;17)(q24.1;q21.2)/PML::RARA	≥ 10%^5^		t(15;17)(q24.1;q21.2)/PML::RARA			
	APL with other RARA rearrangements	≥ 10%^5^		t(1;17)(q42.3;q21.2)/IRF2BP2::RARA; t(5;17)(q35.1;q21.2)/NPM1::RARA; t(11;17)(q23.2;q21.2)/ZBTB16::RARA; cryptic inv(17q) or del(17)( q21.2q21.2)/STAT5B::RARA, STAT3::RARA; Other genes rarely rearranged with RARA:TBL1XR1 (3q26.3), FIP1L1 (4q12), BCOR (Xp11.4)			
AML with *RUNX1*::*RUNX1T1* fusion	=	≥ 10%^5^		t(8;21)(q22;q22.1)/RUNX1::RUNX1T1			
AML with *CBFB*::*MYH11* fusion	=	≥ 10%^5^		inv(16)(p13.1q22) or t(16;16)(p13.1;q22)/CBFB::MYH11			
AML with *DEK*::*NUP214* fusion	=	≥ 10%^5^		t(6;9)(p22.3;q34.1)/DEK::NUP214			
AML with *RBM15*::*MRTFA* fusion	Included in "AML with other rare recurring translocations"	≥ 10%^5^		t(1;22)(p13.3;q13.1)/RBM15::MRTF1			
AML with *BCR*::*ABL1* fusion	=	≥ 20%^6^		t(9;22)(q34.1;q11.2)/BCR::ABL1			
AML with *KMT2A* rearrangement	AML with t(9;11)(p21.3;q23.3)/MLLT3::KMT2A	≥ 10%^5^		t(9;11)(p21.3;q23.3)/MLLT3::KMT2A			
	AML with other KMT2A rearrangements	≥ 10%^5^		*Includes AMLs with: t(4;11)(q21.3;q23.3)/AFF1::KMT2A; t(6;11)(q27;q23.3)/AFDN::KMT2A; t(10;11)(p12.3;q23.3)/MLLT10::KMT2A; t(10;11)(q21.3;q23.3)/TET1::KMT2A; t(11;19)(q23.3;p13.1)/KMT2A::ELL; t(11;19)(q23.3;p13.3)/KMT2A::MLLT1 (Occurs predominantly in infants and children)			
AML with *MECOM* rearrangement	AML with inv(3)(q21.3q26.2) or t(3;3)(q21.3;q26.2)/GATA2; MECOM(EVI1)	≥ 10%^5^		inv(3)(q21.3q26.2) or t(3;3)(q21.3;q26.2)/GATA2; MECOM(EVI1)			
	AML with other MECOM rearrangements	≥ 10%^5^		Includes AMLs with: t(2;3)(p11~23;q26.2)/MECOM::?; t(3;8)(q26.2;q24.2)/MYC, MECOM; t(3;12)(q26.2;p13.2)/ETV6::MECOM; t(3;21)(q26.2;q22.1)/MECOM::RUNX1			
AML with *NUP98* rearrangement	Included in "AML with other rare recurring translocations"	≥ 10%^5^					
AML with *NPM1* mutation	=	≥ 10%^5^			NPM1		
AML with *CEBPA* mutation	=	≥ 20%^7^			in-frame bZIP CEBPA		
	AML and MDS/AML with mutated TP53	WHO: ≥ 20% blasts in BM or PB	ICC and ELN: MDS/AML if 10-19% blasts in BM or PB; AML if ≥ 20% blasts in BM or PB.		TP53		
AML, myelodysplasia-related	AML and MDS/AML with myelodysplasia-related gene mutations				ASXL1, BCOR, EZH2, SF3B1, SRSF2, STAG2, U2AF1, ZRSR2, RUNX1		
	AML with myelodysplasia-related cytogenetic abnormalities			Complex karyotype (≥3 abnormalities),del(5q)/t(5q)/add(5q), -7/del(7q), +8, del(12p)/t(12p)/add(12p), i(17q), -17/add(17p) or del(17p), del(20q), and/or idic(X)(q13)			
AML, myelodysplasia-related (following a known history of MDS or MDS/MPN)		WHO: ≥ 20% blasts in BM or PB					
AML with other defined genetic alterations	AML with other rare recurring translocations	WHO: ≥ 20% blasts in BM or PB	≥10%	t(1;3)(p36.3;q21.3)/PRDM16::RPN1; t(1;22)(p13.3;q13.1)/RBM15::MRTF1; t(3;5)(q25.3;q35.1)/NPM1::MLF1; t(5;11)(q35.2;p15.4)/NUP98::NSD1; t(7;12)(q36.3;p13.2)/ETV6::MNX1; t(8;16)(p11.2;p13.3)/KAT6A::CREBBP; t(10;11)(p12.3;q14.2)/PICALM::MLLT10; t(11;12)(p15.4;p13.3)/NUP98::KMD5A; NUP98 and other partners; t(16;21)(p11.2;q22.2)/FUS::ERG; t(16;21)(q24.3;q22.1)/RUNX1::CBFA2T3; inv(16)(p13.3q24.3)/CBFA2T3::GLIS2			
Acute myeloid leukaemia, defined by differentiation^8^	AML, NOS						
AML with minimal differentiation		WHO: ≥ 20% blasts in BM or PB	ICC and ELN: MDS/AML if 10-19% blasts in BM or PB; AML if ≥ 20% blasts in BM or PB.	Any except other AML defining cytogenetics	Any except other AML defining mutations	• Blasts are negative (<3%) for MPO and SBB by cytochemistry • Expression of two or more myeloid-associated antigens, such as CD13, CD33, and CD117	
AML without maturation						• ≥3% blasts positive for MPO (by immunophenotyping or cytochemistry) or SBB and negative for NSE by cytochemistry • Expression of two or more myeloid-associated antigens, such as MPO, CD13, CD33, and CD117 • Maturing cells of the granulocytic lineage constitute <10% of the nucleated bone marrow cells	
AML with maturation						• ≥3% blasts positive for MPO (by immunophenotyping or cytochemistry) or SBB by cytochemistry • Maturing cells of the granulocytic lineage constitute ≥10% of the nucleated bone marrow cells • Monocyte lineage cells constitute < 20% of bone marrow cells • Expression of two or more myeloid-associated antigens, such as MPO, CD13, CD33, and CD117	
Acute basophilic leukaemia						• Blasts & immature/mature basophils with metachromasia on toluidine blue staining • Blasts are negative for cytochemical MPO, SBB, and NSE • No expression of strong CD117 equivalent (to exclude mast cell leukemia)	
Acute myelomonocytic leukaemia						• ≥20% monocytes and their precursors • ≥20% monocytes and their precursors • ≥3% of blasts positive for MPO (by immunophenotyping or cytochemistry)	
Acute monocytic leukaemia						• ≥80% monocytes and/or their precursors (monoblasts and/or promonocytes) • <20% maturing granulocytic cells • Blasts and promonocytes expressing at least two monocytic markers including CD11c, CD14, CD36 and CD64, or NSE positivity on cytochemistry	
Acute erythroid leukaemia						• ≥30% immature erythroid cells (proerythroblasts) • Bone marrow with erythroid predominance, usually ≥80% of cellularity	
Acute megakaryoblastic leukaemia						• Blasts express at least one or more of the platelet glycoproteins: CD41 (glycoprotein llb), CD61 (glycoprotein IIIa), or CD42b (glycoprotein lb)	
Myeloid Sarcoma	=	Absence of bone marrow involvement		Any	Any	Any	
Myeloid neoplasms post cytotoxic therapy							
Myeloid neoplasms associated with germline predisposition							

^1.^Khoury J, et al, 5th Edition WHO Classification of Haematolymphoid Tumours: Myeloid and Histiocytic/Dendritic Neoplasms. Leukemia 2022; https://doi.org/10.1038/s41375-022-01613-1. ^2.^ Arber D, et al, The International Consensus Classification (ICC) of Myeloid Neoplasms and Acute Leukemias: Integrating Morphological, Clinical, and Genomic Data. Blood 6/14/2022. ^3.^ ﻿Döhner H, Wei AH, Appelbaum FR, et al. Diagnosis and Management of AML in Adults: 2022 ELN Recommendations from an International Expert Panel. Blood. 2022; ^4.^ Only used as modifiers in the ICC and ELN 2022 classification systems. ^5.^ The WHO does not require any specific blast percentage cutoff for a diagnosis of AML in the presence of AML-defining genetic abnormalities. ^6.^ AML with t(9;22)(q34.1;q11.2)/BCR::ABL1 requires bone marrow or peripheral blood blast count of ≥20% due to overlap with progression of chronic myeloid leukemia, BCR::ABL1-positive. ^7.^ AML with and biallelic CEBPA mutations requires bone marrow or peripheral blast count count of ≥20%. ^8.^ Shared diagnostic criteria include: 1) ≥ 20% blasts in bone marrow and/or blood (except for acute erythroid leukaemia); Criteria for AML types with defined genetic alterations are not met; Criteria for mixed-phenotype acute leukemia are not met (relevant for AML with minimal differentiation); Not fulfilling diagnostic criteria for myeloid neoplasm post cytotoxic therapy; No prior history of myeloproliferative neoplasm. Abbreviations: APL Acute promyelocytic leukemia, AML Acute myelocytic leukemia, BM bone marrow, MPO myeloperoxidase, NSE nonspecific esterase, PB peripheral blood, SBB Sudan Black B.

Genetic features increasingly supersede blast percentage in the distinction and classification of MDS and AML as well. In the 2008 WHO classification, the presence of *RUNX1*::*RUNX1T1*, *CBF*::*MYH11, PML*::*RARA* alone was sufficient to make a diagnosis of AML (or in the case of the latter, APL), regardless of the blast percentage. In the most recent 2022 edition, this exception now extends to all AML subtypes with recurrent genetic abnormalities including AML with t(9;11)(p21.3;q23.3)/*MLLT3::KMT2Ac*, t(6;9)(p22.3;q34.1)/*DEK::NUP214*, inv(3)(q21.3q26.2) or t(3;3)(q21.3;q26.2)/*GATA2, MECOM(EVI1)*, other rare recurring translocations, and mutated *NPM1*. While the ICC still requires a blast percentage of ≥ 10% in the setting of a genetically-defining lesion, the WHO does not include a lower limit in its definition of genetically-defined AML.

Similarly, the finding of dysplasia has lost some significance in the most recent ICC system. Historically, MDS required the presence of dysplasia in at least 10% of a single lineage; cases of cytopenias and clonal genetic abnormalities without dysplasia have more recently been classified as CCUS. The most recent ICC classification system, however, allows for the diagnosis of MDS, even without a finding of dysplasia if 5q deletion, monosomy 7/7q deletion, complex karyotype, or multi-hit TP53 are found. Furthermore, the finding of multilineage dysplasia is no longer used to distinguish *de novo* AML from secondary AML. AML is now either classified as myelodysplasia related on the basis of specific genetic abnormalities or may be amended by the diagnostic qualifiers “progression from MDS” or “progression from MDS/MPN.”

The emphasis of genetics over morphology is particularly notable in the ICC classification of TP53-mutated myeloid neoplasms, which are now grouped together and set apart from other MDS and AML subtypes in order to convey the uniquely aggressive nature of these diseases, regardless of the blast percentage or presence of dysplasia.

Overall, the new classification schemas reflect the understanding that genetics determine disease behavior, which in turn correlates with blast percentage. Thus, MDS’ with indolent behavior, such as isolated 5q deletion or *SF3B1* rarely presents at diagnosis with excess blasts, whereas MDS with AML-like mutations (*DNMT3A, NPM1, FLT3, IDH1*, and *RUNX1*) almost exclusively presented with excess blasts (54).

In the absence of informative genetic data, blast percentage is the next best surrogate for disease behavior. In a retrospective analysis of 2,043 patients with MDS, Bersanelli et al. used Bayesian networks and Dirichlet processes to reclassify MDS using demographic data, clinical features, and genetic characteristics (54). Overall, clinical characteristics explained 42% (95% CI 34%-54%) of the variability in overall survival, with blast percentage being the largest factor amongst all clinical characteristic considered. In contrast, the percentage of variability explained by genetic factors were gene mutations 13% (95% CI 8-24%), chromosomal abnormalities 4% (95% CI 2-8%), and gene-gene interactions 3% (95% CI 0-8%). For patients in whom the genetic data does not provide prognostic clarity, the blast percentage may still serve as a proxy for genetic or epigenetic features that are present but have not yet been characterized. Blast percentage is therefore still used to distinguish MDS from AML, but the introduction of a new entity within the ICC classification system, MDS/AML, which includes all myeloid neoplasms without defining genetic lesions and 10-19% blasts, softens the distinction and acknowledges the fact that these diseases exist on a continuum.

Nonetheless, the optimal blast threshold remains a source of controversy. In defense of the original FAB cutoff of 30%, a retrospective analysis of 1652 patients found that patients with 20-29% blasts (previously classified as refractory anemia with excess blasts in transformation) were more similar to patients with MDS (<20% blasts) in terms of their clinicopathology, molecular characteristics, and outcomes than they were to patients with ≥ 30% blasts (55). On the other hand, others have found comparable outcomes between AML and MDS-EB2 (56, 57). In another analysis comparing patients with MDS-EB2 with complex karyotype and *TP53* mutation to patients with AML and similar genetic characteristics, the two groups were largely indistinguishable and had uniformly poor outcomes regardless of blast count (58).

While the use of a rigid threshold to define MDS and AML seems to compromise the nuanced conception of MDS and AML as representing sides of a spectrum of disease, it also has practical benefits. A blast threshold is used for trial enrollment to ensure a trial is enrolling the target population; it is helpful in providing guidance to community practitioners when deciding between the use of an MDS vs. AML treatment paradigm; and as a means for providing clinical annotation for translational research efforts. To balance the need for nuance and discrete disease categories, the term MDS/AML has been adopted by the European Leukemia Network and the International Consensus Classification to denote myeloid neoplasms with 10-19% peripheral or bone marrow blasts (59, 60). A welcomed consequence of this change might be the inclusion of patients with MDS/AML, as defined in the new ICC guidelines, into clinical trials that would otherwise have been restricted to either MDS or AML (57).

## The practical importance of understanding the relationship between MDS and AML

An understanding of clonal evolution and the genetics of MDS and AML is critical for informed clinical decision making at the bedside. Clinical treatment paradigms have been designed to fit an either-or binary, but now must increasingly be adapted to a more nuanced biology-driven understanding of these diseases. Expectation management, deciding whether to pursue an MDS or an AML-oriented treatment paradigm, choosing the most appropriate AML therapy within an AML paradigm, and the decision of whether or not to pursue transplant all hinge on an understanding of a myeloid neoplasm’s past and future clonal trajectories. An extensive baseline characterization of the disease is of course a prerequisite in developing an accurate conception of the disease biology and behavior, but iterative reassessment over time is also crucial to the process of defining the disease. In patients without overt AML, there isn’t necessarily a penalty to deferring treatment (61), so providers should be patient and methodical, gathering more information over time if necessary, in defining the contours of the disease and in formulating the optimal treatment plan. Increasingly, measurable residual disease (MRD) is of interest for AML (and MDS, to a lesser extent) therapeutic optimization (62–64). This does requires an ongoing understand of the details of the sAML and what MDS-associated mutations were present prior to AML.

## Diagnosing AML masquerading as MDS: AML with < 20% blasts

Infrequently, a case of AML may present with a bone marrow blast percentage < 20%, contradicting the historical rule of thumb used for distinguishing MDS and AML. Unlike their true MDS counterparts, these cases typically lack the tell-tale dysplasia seen in MDS and they will have mutations that are otherwise exclusively seen in AML such as *RUNX1*::*RUNX1T1*, *CBF*::*MYH11*, or mutated *NPM1*. These patients have demographic and disease characteristics similar to patients with *de novo* AML, and the disease behavior is also more akin to that of *de novo* AML. They are also responsive to AML-type therapy, regardless of blast percentage at presentation (65–67). The presence of these mutations should lead to clinicians to consider whether the disease may be biologically and behaviorally more consistent with AML. Overreliance on morphology and a failure to recognize that genetically, these cases are more consistent with AML than MDS, might lead to misguided discussions regarding prognosis, the need for allogeneic hematopoietic cell transplant (alloHCT), and the adoption of an MDS-based therapeutic paradigm. Recognizing that biologically and behaviorally these cases are actually most consistent with favorable risk AML leads to an entirely different prognostic outlook and therapy plan in which intensive chemotherapy may very well be curative. The precedence of genetics over morphology in such cases has now been codified in the most recent WHO diagnostic guidelines (12).

## Acknowledging the harbingers of progression from MDS to AML

One of the most feared outcomes of MDS, with or without therapy, is progression to sAML. Survival after progression is short, and treatment outcomes for sAML are inferior to those for *de novo* AML (68, 69). The risk of progression is therefore used to inform prognosis and to guide clinical decision making; aggressive therapies, such as hypomethylating agents and alloHCT, are typically reserved for patients with a high risk of progression to sAML and poorer overall prognosis. Historically, the risk of progression has been estimated on the basis of the depth of cytopenias, the percentage of bone marrow blasts, and cytogenetic characteristics (70, 71), however, more recent studies have demonstrated that somatic gene mutations are also powerful, independent predictors of both progression to sAML and mortality. MDS with mutations in genes such as *TP53, CBL, EZH2, RUNX1, U2AF1*, and *ASXL1* are more likely to progress to sAML than expected based on traditional scoring systems alone, whereas *SF3B1* is associated with a lower risk of progression (72–77). Of course, mutations frequently co-occur and the complex interactions between them frustrates efforts to integrate these factors into prognostic scoring systems, though a better understanding of them also promises to lead towards more precise prognostication and treatment. Clinical reassessment for patients with MDS is suggested at times of clinical change such as falling blood counts, increasing systemic symptoms, or recurrent infection which likely are precipitated by changing disease biology and possible progression to AML. Surveillance bone marrow assessments are advocated by some but not routinely preformed in clinical practice.

Close attention to the genetic evolution of the disease over time can alert a provider to the imminent transformation to AML. The acquisition of new gain-of-function mutations in signaling genes (*FLT3, WT1, PTPN11, NRAS*), *NPM1*, or *IDH1/2* in the setting of MDS heralds transformation to AML (49, 73). In some cases, mutations in signaling pathway genes such as *NRAS* and *PTPN11*, as well as chromatin regulators such as *ASXL1* and *EZH2*, can be seen at low VAFs in the MDS state. The presence of these “second hits,” even at very low VAFs is associated with a higher risk of transformation than would be predicted by standard prognostic scoring systems (9, 48, 78). In the course of progression to sAML, the allelic burdens of these mutations rise, reflecting an expansion of these subclones, which drives the transformation to sAML (79). This transformation can occur rather rapidly over the course of weeks to months. Therefore, monitoring for newly acquired mutations and close attention to changing allelic burdens can be informative and guide expectation management and decision making. The presence of a new gain-of-function mutation may also reveal new avenues of treatment. The mutations that tend to precipitate transformation to AML are gain-of-function mutations that are more amenable to FDA-approved targeted therapies such as FLT3 inhibitors and IDH1/2 inhibitors (80). Finally, recognition of a new mutation heralding the onset of AML can prompt a more rapid adoption of an AML treatment paradigm and pursuit of curative alloHCT.

Another illustrative example is that of MDS with an isolated del 5q (one other abnormality other than del 7q is allowed), which is often referred to as 5q minus syndrome. This form of MDS has a distinctive clinical phenotype characterized by a predominant anemia, normal or even increased platelets, few blasts with a low risk of transformation to AML, and a high rate of responsiveness to lenalidomide (41). In most cases, patients with 5q minus syndrome have relatively long overall survival when managed with a combination of supportive care and lenalidomide and therefore, attempting a curative approach alloHCT is generally unlikely to prolong survival. A subset, however, may present with or acquire, as a result of natural history or selective pressure from lenalidomide, a “second-hit” mutation in *TP53*. Whenever it is present, a coincident *TP53* mutation is associated with an increased risk of progression to sAML. In clinical practice, surveillance and testing for new *TP53* mutations, particularly in the setting of a loss of response to lenalidomide, can foreshadow transformation and may serve as a trigger to pursue curative alloHCT in an otherwise indolent disease.

## Classifying secondary AML

As previously noted, the definition of secondary AML has come to rest largely on the genetic features of the disease. In the most recent WHO and ICC classification systems, sAML (referred to as AML, myelodysplasia-related) is defined as AML with defining genetic abnormalities such as unbalanced loss of chromosomal material and mutations in genes involved in the spliceosome. In the WHO system, patients with AML and a known history of MDS or MDS/MPN are classified together with those patients with AML and MDS-related genetic abnormalities, while in the 2022 ICC classification system, a history of MDS or MDS/MPN is denoted as a diagnostic qualifier. In both the WHO and ICC, the finding of dysplasia in AML is no longer used to distinguish *de novo* and secondary AMLs.

Properly distinguish sAML from *de novo* AML is a clinically important task. sAML is well-known to be associated with lower rates of complete remission and worse overall survival than *de novo* AML (68, 69). This is due in part to the greater underlying intratumoral heterogeneity of the disease, which increases the likelihood of primary resistance to any given drug regimen and increases the likelihood of developing secondary resistance. Outcomes in sAML are also affected by prior MDS-directed therapy. Patients who develop sAML after prior HMA treatment have particularly poor outcomes. The treatment of sAML is made more complicated by the coexistence of transformed AML and residual MDS subclones. Treating sAML is therefore like treating two co-existing diseases. AML-directed therapies may be effective against the AML subclone, but not the antecedent MDS. Treatment with AML-directed therapy may therefore eliminate the AML, but still result in relapse of the initial MDS, which in turn, may progress once again. Similarly, MDS-directed therapies, take the example of lenalidomide in 5q minus syndrome, are not effective by themselves in the treatment of sAML bearing a 5q deletion due to the presence of transformed subclones that are no longer entirely dependent on the advantages of the 5q deletion for survival. There is some data to support the use of conventional 7 + 3 (cytarabine and an anthracycline) in combination with lenalidomide for such cases, but it is limited to phase 2 data (81). Thus, patients with sAML who are relatively fit should be recommended for alloHCT to mitigate the increased risk of relapse associated with antecedent MDS.

Patients with sAML also derive greater benefit from treatment with a new liposomal formulation of conventional cytarabine and daunorubicin (“7+3”) called CPX-351. In a phase 2 study, outcomes were most improved in the subset of patients with secondary and treatment-related AML (82), leading to the completion of a randomized phase 3 trial of CPX-351 vs. 7 + 3 in patients with newly-diagnosed, untreated secondary and therapy-related AML (83). Patients in treated on the CPX-351 arm had significantly higher overall remission rates (47.7% v 33.3%; two-sided P = .016) and median overall survival (9.56 v 5.95 months; hazard ratio, 0.69; 95% CI, 0.52 to 0.90; one-sided P = .003). Furthermore, in a subset analysis, outcomes for patients who underwent alloHCT after achieving complete remission with 7 + 3 vs. CPX-351 were compared (84). Patients who achieved CR with CPX-351 had decreased rates of relapse and better overall survival compared to patients who had previously received 7 + 3, hinting at the possibility that CPX-351 leads to deeper remissions. These data provide further support for the use of CPX-351 over 7 + 3 in older patients with sAML. The excitement surrounding CPX-351 is tempered by the cost of the drug (85), and recent data suggesting that outcomes with CPX-351 are similar to those with HMA/Ven, a regimen that is considered non-intensive (86–88). Furthermore, it does not appear to be more effective than 7 + 3 in patients with biallelic TP53-mutated AML, the subset of sAML patients with the worst outcomes.

## Higher risk MDS vs. AML

As previously noted, the bone marrow blast percentage cut off that distinguishes MDS from AML in cases without AML-defining genetic features remains at 20%. We can acknowledge the need for such a cutoff without being dogmatic. There are some cases in which a patient with properly categorized high risk MDS, particularly those with excess blasts, may benefit from AML-type therapy such as intensive chemotherapy or venetoclax-based therapy. Some centers routinely consider patients with MDS-EB2 eligible for AML-type therapy (57) and intensive chemotherapy remains an option for higher risk MDS according to the NCCN guidelines (89). Although there are some patients who may benefit from such an approach, there is no reliable strategy for identifying these patients and outcomes with intensive chemotherapy in this context are poor. It is not yet known whether use of CPX-351, a novel liposomal formulation of conventional cytarabine and daunorubicin (“7+3”) that is approved for use in sAML, in this setting might yield better results (90).

Venetoclax-based therapy, on the other hand, has demonstrated acceptable safety and efficacy in early phase trials of untreated higher risk MDS, albeit dose-reduced compared to AML dosing (91). There is not yet enough evidence to support the routine use of azacitidine and venetoclax in the upfront setting, but the ongoing phase 3 Verona trial (NCT04401748), which compares azacitidine monotherapy with azacitidine plus venetoclax in patients with higher-risk MDS (defined as fewer than 20% bone marrow blasts, overall IPSS-R greater than 3) should provide more definitive data. Although the use of HMA/Ven in the upfront setting is still being investigated, there is data to support the addition of venetoclax to an HMA in patients who have not responding to HMAs alone (92–94). In patients who had not responded to ≥ 4 cycles of HMA, salvage HMA/Ven resulted in a 44% overall response rate and a median overall survival of 11.4 months (95% CI 5.7 to not estimable). It is important to recognize in these circumstances that the optimal duration of venetoclax in this patient population has not been identified. Furthermore, these patients are prone to developing profound and prolonged aplasias that require close monitoring, likely due to the fact that most of the patient’s residual hematopoiesis is derived from the MDS clone that is being targeted.

## Biallelic loss of *TP53*: When the MDS vs. AML label may no longer matter at all

*TP53* loss-of-function mutations are common in both MDS and AML, but their clinical significance depends largely upon whether the loss of function is monoallelic, biallelic (BiTP53), or is accompanied by a complex karyotype (CK). Biallelic loss of function may result from the acquisition of multiple point mutations or a single point mutation on one allele combined with loss of genetic material from 17p on the other allele resulting in copy neutral loss of heterozygosity. Patients with either MDS or AML with BiTP53 or CK-TP53mut have similarly dismal outcomes. In contrast, patients with monoallelic TP53 mutation without CK have outcomes that are intermediate between that of BiTP53/CK-TP53mut and Non-CK-TP53wt (95, 96). BiTP53 and CK-TP53mut myeloid neoplasms are poorly responsive to both MDS and AML-type treatment paradigms (97, 98) and have poor outcomes even with alloHCT (99, 100).

Distinguishing between MDS and AML in these cases doesn’t seem to be of much biological or clinical importance; regardless of where they fall on the MDS-AML spectrum or treatment paradigm, the disease biology, patient characteristics, and outcomes are similar. Patients with BiTP53/CK-TP53mut myeloid neoplasms should therefore be prioritized for treatment on clinical trials that specifically target their unique biology. Promising novel agents currently under investigation in this patient population include those that target the CD47-signal regulatory protein alpha (SIRPα) interaction and agents engineered to restore the function of mutated TP53 proteins. CD47 is an immune checkpoint cell surface marker that is upregulated in high risk MDS and AML myeloblasts that binds SIRPα on circulating immune cells including macrophages, sending a “don’t eat me” signal that inhibits phagocytosis. Blocking this interaction through anti-CD47 (magrolimab) or SIRPα antibodies has shown promising efficacy in early phase studies, particularly in TP53-mutated myeloid neoplasms (101). APR-246 (eprenetapopt) is first-in-class small molecule that induces apoptosis in TP53-mutated neoplasms by compelling mutant-TP53 protein into a functional conformation, thereby restoring activity (102). In a phase 2 study of patients with TP53-mutated MDS and AML, APR-246 plus azacitidine had a side-effect profile similar to that which is expected with azacitidine monotherapy and resulted in an overall response rate of 71% with 44% achieving CR and a median OS of 10.8 months (103). Unfortunately, in a preliminary analysis, the phase 3 study comparing APR-246 and azacitidine and azacitidine alone did not meet its primary endpoint of complete remission rate (104). Nonetheless, the concept still holds promise and further investigation may reveal a role for APR-246 or a related, second generation agent to target mutated TP53.

## MDS/AML agnosticism in clinical trials

Greater emphasis on drug development that targets the specific genetic drivers of disease will necessitate further relaxation of the boundaries that distinguish MDS from AML (57, 103, 105). Not only does it make biological sense to include patients with either MDS or AML when testing a targeted agent against a shared driver mutation, but it is practical; these genetic targets occur in small enough subsets of patients that it may be necessary for accrual. In turn, the development of drugs that are agnostic to the distinction of MDS and AML will further de-emphasize the clinical relevance of this distinction.

## Conclusion

MDS and AML are inextricably intertwined through their shared pathogenesis, overlapping clinical features, and MDS’s predilection for transformation. Appropriate treatment of these entities requires an expertise with the biological characteristics of each and how these characteristics shape the treatment paradigms that come to be associated with each of them.

## Author contributions

AA and AD were responsible for the conception and writing of this manuscript. All authors contributed to the article and approved the submitted version.

## Conflict of interest

The authors declare that the research was conducted in the absence of any commercial or financial relationships that could be construed as a potential conflict of interest.

## Publisher’s Note

All claims expressed in this article are solely those of the authors and do not necessarily represent those of their affiliated organizations, or those of the publisher, the editors and the reviewers. Any product that may be evaluated in this article, or claim that may be made by its manufacturer, is not guaranteed or endorsed by the publisher.

## Abbreviations

MDS, myelodysplastic neoplasms; AML, acute myeloid leukemia; WHO, World Health Organization; CHIP, Clonal Hematopoiesis of Indeterminate Potential; CCUS, Clonal Cytopenias of Undetermined Significance; sAML, secondary AML; MDS-RS, MDS with ring sideroblasts; VAF, variant allele frequency; FAB, French-American-British; MDS-EB, MDS-excess blasts; alloHCT, allogeneic; hematopoietic cell transplant.
